# A study of the interference colors of the microscopic textures simulated along the lyotropic phase sequence: cholesteric discotic–cholesteric biaxial–unwound cholesteric calamitic

**DOI:** 10.1140/epje/s10189-026-00593-9

**Published:** 2026-06-01

**Authors:** D. D. Lüders, E. Akpinar, G. E. Delmanoco

**Affiliations:** 1 Departamento de Física, State University of Maringá, Maringá, Brazil; 2 Department of Chemistry, Faculty of Arts and Sciences, Bolu Abant Izzet Baysal University, 14030 Golkoy, Bolu, Türkiye

## Abstract

The interference colors of the microscopic textures, observed under a polarized microscope for the oriented cholesteric discotic (Ch_D_)–cholesteric biaxial (Ch_B_)–unwound cholesteric calamitic (N_C_^*^) phases sequence in the presence of a magnetic field, were studied in this work. The referred textures were interpreted from both experimental and theoretical views. The lyotropic nematic host mixtures consisted of potassium laurate (KL), 1-undecanol (unDeOH), potassium sulphfate (K_2_SO_4_), and water (H_2_O). The chiral guest molecule brucine was added to the host nematic mixture to induce the cholesteric phases. The refractive indices and birefringences of the lyotropic samples were measured along the nematic calamitic (N_C_)–nematic biaxial (N_B_)–nematic discotic (N_D_) phases sequence as a function of temperature, and they were used to simulate the stripe textures of the oriented cholesteric phases, Ch_D_ and Ch_B_, theoretically. It was shown that the experimental and simulated results are in good agreement with each other.

**Graphical abstract:**

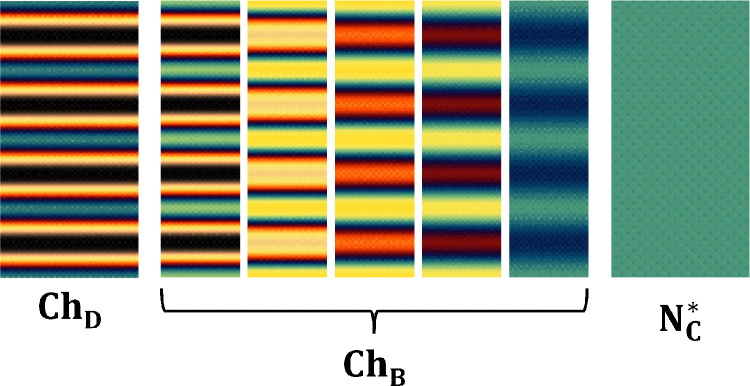

## Introduction

Lyotropic liquid crystals are micellar systems obtained from mixtures of surfactants and solvents, for instance, detergents and water [1]. In most cases, salts and cosurfactants, such as long-chain alcohols, are added to these mixtures [2–7]. The concentrations of the constituents of the lyotropic samples affect the mesophase structures. In this sense, several phase diagrams were proposed by researchers [8–13]. Among them, in 1980, Yu and Saupe reported a first phase diagram [8], in which the biaxial nematic phase (N_B_) was recognized for the first time between two uniaxial nematic ones: discotic nematic (N_D_) and calamitic nematic (N_C_). In their work, the biaxial nematic phase symmetry was considered as orthorhombic, where the three principal axes are mutually orthogonal.

Following the discovery of the N_B_ phase, several controversies arose in the literature regarding its existence due to the lack of a suitable model to explain its formation mechanism. Eventually, Figueiredo Neto et al. proposed an Intrinsically Biaxial Micelle model (IBM) to explain microscopically the origin of not only biaxial but also uniaxial nematic phases [14]. In this model, it was assumed that the micelles have an orthorhombic symmetry in the three nematic phases, and different orientational fluctuations around the symmetry axes fixed on the micelles are responsible for the formation of these phases. In addition, the Refs. [15, 16] show how the N_B_ phase in lyotropic systems can be stabilized from the experimental conditions point of view.

The lyotropic nematic phases can be aligned in the presence of an external magnetic field due to the preferred alignment of their phase directors with respect to the magnetic field direction [17]. The N_D_ and N_C_ phases exhibit two diamagnetic susceptibilities (${\chi}_{e}$ and ${\chi}_{o}$), and they are characterized by negative and positive anisotropies of diamagnetic susceptibility $(\Delta \chi )$, respectively, where $\Delta \chi ={\chi}_{e}-{\chi}_{o}$. On the other hand, the N_B_ phase has three diamagnetic susceptibilities, ${\chi}_{1}>{\chi}_{2}>{\chi}_{3}$, and its anisotropy is given by $\Delta \chi ={\chi}_{3}-({\chi}_{1}+{\chi}_{2})/2$ [10, 13]. When the component ${\chi}_{3}$ is larger than the average values of the other two components of the diamagnetic susceptibilities, the biaxial phase is considered diamagnetically positive; otherwise, negative.

From the optic point of view, N_D_ and N_C_ phases are considered as uniaxial media, i.e., they have a unique optic axis. For the N_D_ (N_C_) phase, the optic axis aligns perpendicular (parallel) to the applied magnetic field direction [17], and it is optically positive (negative) [18–21]. On the other hand, the two optic axes of the N_B_ phase belong to a plane parallel to the magnetic field $\overrightarrow{\mathrm{H}}$ [22]. The angle between these two axes is denoted by 2V [23], and it depends on the temperature [22, 24]. Similar to uniaxial phases, the biaxial nematic phase is considered as optically positive when ${n}_{3}-{n}_{2}>{n}_{2}-{n}_{1}$, optically negative for ${n}_{3}-{n}_{2}<{n}_{2}-{n}_{1}$, and optically neutral (without sign or undetermined) when ${n}_{3}-{n}_{2}={n}_{2}-{n}_{1}$, where ${n}_{1}$*,* ${n}_{2}$ and ${n}_{3}$ are refractive indices [23, 25, 26].

The nematic phases can be modified by the addition of chiral dopants to obtain cholesteric counterparts [27–30]. A minimum concentration of the chiral molecules is necessary to induce cholesteric phases from the host nematic ones [31]. The structure of the cholesteric phase can be considered as a twisted nematic phase [32, 33]. In this structure, the micelles are arranged along with a preferred orientation in planes, each of which is rotated by an angle with respect to the other layers. In other words, it is assumed that the nematic director is continuously twisted to form a supermolecular helicoidal structure in a cholesteric phase due to the transfer of the chirality of the chiral dopant molecule to the whole nematic host phase [34]. Going from one plane to another, the director undergoes a continuous rotation around one axis, known as the cholesteric axis or helix axis. The distance traveled along the cholesteric axis to complete a full rotation (360°) of the micelles around the quoted axis defines the pitch length of the cholesteric phase, and its length is dependent on the mole concentration of the chiral dopants and, in general, temperature [27, 31, 35–38].

The cholesteric axes of the discotic cholesteric, Ch_D_, and biaxial cholesteric, Ch_B_, phases align in a direction parallel to the magnetic field direction, and, on the other hand, the cholesteric structure of the calamitic cholesteric, Ch_C_, phase is unwound by the effect of the magnetic field [30, 39–41]. In this last case, the Ch_C_ phase is transformed into a nematic calamitic (N_C_^*^) one [41]. From the optic point of view, the cholesteric axis corresponds to the optic axis of the Ch_D_, Ch_B,_ and Ch_C_ phases [31, 42, 43]. Macroscopically, the Ch_D_ (Ch_C_) phase is optically negative (positive) [42], while the Ch_B_ phase in the presence of the magnetic field is only negative [44]; otherwise, microscopically (on each nematic plane), the optic sign is defined by the nematic phases: N_D_, N_B_, and N_C_ [44, 45].

From the microscopic textures observed under a polarizing optical microscope, the interference colors exhibited by the oriented nematic phase sequence, N_D_–N_B_–N_C_, were carefully described in our recent study [44]. Now, in the present work, we aim to extend our investigation to interpret the interference colors obtained from the oriented cholesteric phase sequence, Ch_D_–Ch_B_–N_C_^*^, microscopically. Here, the interference colors of each cholesteric stripe will be interpreted and discussed considering the results of the refractive index measurements in nematic phases. Besides, the stripes of the oriented Ch_D_ and Ch_B_ phases will also be generated theoretically. We highlight that no other work was reported in the literature proposing an interpretation for the interference colors exhibited by cholesteric phases investigated under polarizing microscope. Furthermore, this study supports the literature with some experimental data, and exhibits some new scientific outputs arising from both (i) the measurements of the refractive indices of the nematic phases, especially along the biaxial one, to generate cholesteric counterparts, and (ii) the interference colors showing a certain similarity between experimental and simulated results, considering the microscopic results and the laser conoscopy results obtained from cholesteric and nematic phases, respectively. In particular, the latter distinguishes this study from others in the literature in terms of methodology.

## Fundamental

Crystallography theory is essential for studying anisotropic media, such as Liquid Crystals and Crystals [23, 25]. It is known that when the light propagates inside these media, one may suffer a double refraction phenomenon, which is related to the separation of the incident ray (wave) into two rays [46]. These new rays are mutually perpendicular and polarized to each other, and they propagate inside the medium with two different velocities $(v=c/n)$, each associated with one refractive index $(n)$. Besides, these rays are also known as slow and fast waves in association with their velocities, which are restricted by higher and lower refractive indices, respectively.

The optical properties of the crystals may be described by the optical indicatrix [23, 25, 26]. The three-dimensional ellipsoid that represents the indicatrix is given by

1 $$\frac{{x}^{2}}{{n}_{1}^{2}}+\frac{{y}^{2}}{{n}_{2}^{2}}+\frac{{z}^{2}}{{n}_{3}^{2}}=1$$

where ${n}_{1},{n}_{2},$ and ${n}_{3}$ are the principal refractive indices, and they correspond to the main semi-axes of the ellipsoid, being along the x, y, and z axes, respectively. In addition, these semi-axes may or may not coincide with the crystallographic directions for some classes of the biaxial crystals [23, 25, 47].

For the lyotropic liquid crystals, the optical indicatrix may have three representations [23]: (i) Isotropic phase is represented by a sphere, with ${n}_{1}={n}_{2}={n}_{3}$; (ii) Uniaxial nematic phases, N_D_ and N_C_, are characterized by having two refractive indices equal and the third one different, e.g., ${n}_{1}={n}_{2}\ne {n}_{3}$. In this case, the optical indicatrix is represented by an ellipsoid with a rotational symmetry around the optic axis; (iii) Biaxial nematic phase is characterized by having an orthorhombic and two-fold symmetries, and its optical indicatrix is described by a triaxial ellipsoid, having ${n}_{1}\ne {n}_{2}\ne {n}_{3}$.

**Fig. 1:**
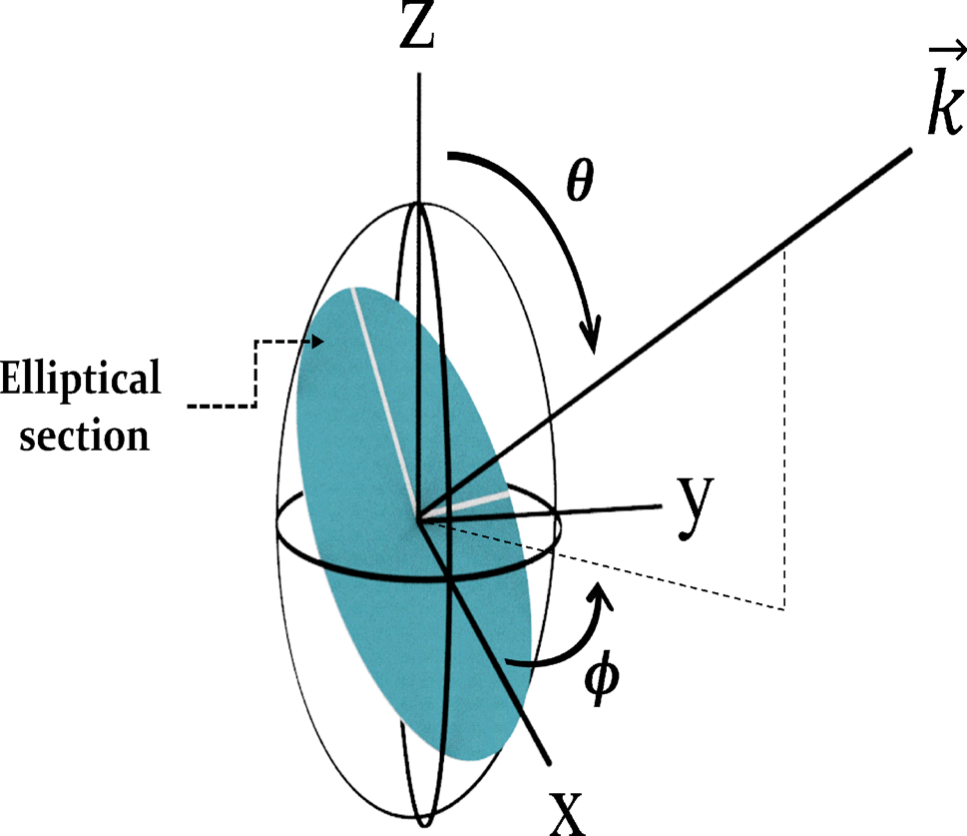
Biaxial indicatrix representation. The normal plane to the output k-vector creates an elliptic section, whose major and minor semi-axes correspond to the polarization of the fast and slow waves, originating from the double refraction of the incident light. The lengths of the semi-axes of the indicatrix along the x, y, and z axes are given by the refractive indices ${n}_{1}, {n}_{2},$ and ${n}_{3}$, respectively

A light ray that propagates inside an anisotropic medium with an arbitrary direction can be represented by the unit k-vector, leaving the optical indicatrix, Fig. 1. An ellipse can be seen from the intersection between the normal plane to the k-vector and the optical indicatrix. The directions of its major and minor axes correspond to two orthogonal polarizations of the two rays originating from the double refraction phenomenon [23]. When the k-vector is made to coincide with the optical axis (or axes) of the uniaxial (biaxial) crystals, the ellipse becomes a circle, and the double refraction does not occur anymore. This is because all the incident wavefronts travel with same velocity inside the crystal, independently of their polarizations, and crystals show an isotropic behavior. In addition, double refraction is not observed when the polarization of the incident light ray coincides with one of the two principal axes of refraction.

When the anisotropic medium is observed through a crossed polarized microscope, the light intensity $(I)$ transmitted by the analyzer is given by [46, 48]:

2 $$I={I}_{o}{\mathrm{sin}}^{2}\left(\frac{\delta }{2}\right),$$

where ${I}_{o}$ corresponds to the light intensity emitted when the polarizers are placed parallel to each other, $\delta$ is the phase difference. For the orthoscopic transmitted light microscope mode, $\delta$ is related to the retardation $(R)$ and wavelength $(\lambda )$ through the relation $\delta =\frac{2\pi }{\lambda }R$. The retardation, given by $R=\Delta n\times d$, is related to the interference colors that can be seen via a polarizing microscope. The relation between observable colors $(R)$, medium thickness $(d)$ and birefringence $(\Delta n)$ is described by the Michel-Lévy interference color chart [23].

When the nematic phases are oriented by the magnetic field applied along the first–third quadrants of the microscopic stage, the semi-axis ${n}_{1}$ of the optical indicatrix of Fig. 1 will be oriented in its direction. On the other hand, the semi-axis ${n}_{2}$ will be induced to align parallel to the stage by the surface. Under crossed polarized microscope observation, the k-vector will be parallel to the z-axis of Fig. 1. Then, under these constraints, it was shown by us in Ref. [49] that the interference colors observed along the oriented N_D_–N_B_–N_C_ phase sequence were associated with the elliptic section of the indicatrix parallel to the microscopic stage, i.e., xy-plane of Fig. 1. Unlike the nematic phases, the optical indicatrix of the cholesteric phases suffers a rotation around the cholesteric or helix axis. In other words, the indicatrix of Fig. 1 suffers a continuum rotation around the semi-axis ${n}_{1}$, continually altering between ${n}_{2}$ and ${n}_{3}$, the semi-axis length along the y-axis [44].

In general, an arbitrary length, $n\left(\theta ,\phi \right)$, between the origin of the indicatrix and its surface can be obtained by rewriting the Eq. (1) in spherical coordinates:

3 $$n\left(\theta ,\phi \right)=\frac{{n}_{1}{n}_{2}{n}_{3}}{\sqrt{{n}_{2}^{2}{n}_{3}^{2}{\mathrm{sin}}^{2}\theta {\mathrm{cos}}^{2}\phi +{n}_{1}^{2}{n}_{3}^{2}{\mathrm{sin}}^{2}\theta {\mathrm{sin}}^{2}\phi +{n}_{1}^{2}{n}_{2}^{2}{\mathrm{cos}}^{2}\theta }} ,$$

where the angles $\theta$ and $\phi$ are specified in Fig. 1.

From Fig. 1, the semi-axes lengths of the indicatrix associated with fast (lower refractive index) and slow (higher refractive index) rays propagating in the anisotropic medium along the z-axis can be determined through Eq. (3). If the experimental configuration of the oriented cholesteric structure is considered, the smallest semi-axis is given by $n\left(\theta =\pi /2,\phi =0\right)={n}_{1}$, while the biggest semi-axis by

4 $$n\left(\theta ,\phi =\pi /2\right)=\frac{{n}_{1}{n}_{2}{n}_{3}}{\sqrt{{n}_{1}^{2}{n}_{3}^{2}{\mathrm{sin}}^{2}\theta +{n}_{1}^{2}{n}_{2}^{2}{\mathrm{c}\mathrm{os}}^{2}\theta }} ,$$

where $\theta =2\pi x/P$, being $x$ some length of the cholesteric axis, and $P$ the cholesteric pitch length. From the Eq. (4), it possible to observe that the higher $\left({n}_{3}\right)$ and lower $\left({n}_{2}\right)$ refractive indices occur for $\theta =0$ and $\theta =\pi /2$, respectively; then, the inequality ${n}_{2}\le n\left(\theta ,\pi /2\right)\le {n}_{3}$ must be satisfied for any value of $\theta$.

In general, for each nematic plane of the cholesteric structure, it is possible to define an effective birefringence associated with the elliptical section onto the xy-plane (Fig. 1) by

5 $$\Delta {n}_{\mathrm{e}\mathrm{f}\mathrm{f}}=n\left(\theta ,\pi /2\right)-{n}_{1} .$$

Equation (5) permits the description of any birefringence of the nematic and cholesteric (cholesteric planes) phases oriented along the x-axis of Fig. 1, i.e., along the first–third quadrants of the microscopic stage. This equation will be fundamental to understand the colors of the microscopic textures obtained along the Ch_D_–Ch_B_–N_C_^*^ phases sequence, once it is related to the retardation, $R=\Delta {n}_{\mathrm{e}\mathrm{f}\mathrm{f}} d$, for a given thickness $d$.

## Experimental

The molar concentrations (in mole fractions) of the lyotropic mixtures exhibiting nematic and cholesteric phases are shown in Table 1. These samples were prepared following two lines from the phase diagrams proposed by Akpinar et al. [31, 50]. Surfactant potassium laurate (KL) was synthesized by following the procedure described in Ref. [51]. Potassium sulfate (K_2_SO_4_), brucine, and 1-undecanol (undeOH) were purchased from Sigma-Aldrich.

**Table 1:** Composition of the lyotropic mixtures (nematic and cholesteric) giving in mole fractions of the potassium laurate (KL), potassium sulfate (K_2_SO_4_), 1-undecanol (undeOH), mili-Q water (H_2_O) and Brucine

Sample	KL	K_2_SO_4_	UndeOH	H_2_O	Brucine	Pitch length ($\upmu$m)	Phase sequence
Nematic	0.0383	0.0060	0.0114	0.9443	–	–	N_C_–N_B_–N_D_
Cholesteric	0.0382	0.0060	0.0114	0.9439	0.0005	150	Ch_C_–Ch_B_–Ch_D_

The lyotropic mixtures were prepared by weighing the constituents in glass test tubes using a Mettler Toledo AT 201 balance with a precision of $\pm 0.01$ mg. The test tubes were closed with their caps and then sealed with parafilm to avoid water evaporation from the tubes. The mixtures were homogenized by applying vortexing and centrifuging occasionally. To improve the alignment of the nematic and cholesteric samples in a magnetic field, water-based ferrofluid was added to the mixture in a proportion of ~ 1 $\mu \mathrm{L}$ per gram of the sample.

The phase sequences in nematic and cholesteric samples were investigated with a polarized light microscope (Leica DML). For analyzing the textures of the samples, some parts of the samples were transferred into 200 $\mu$m thick microslides, which were commercially available from VitroCom. Their ends were sealed with a specific photopolymer, onto which UV light was applied, to prevent water loss. To determine the phase transition temperatures along the phase sequence, the microslides were put into a hot-stage (Instec, MK 1000), which was coupled to the microscope stage, to control temperature. The sample was oriented along the longer length of the microslides via two neodymium magnets. The direction of the magnetic field with a strength of about 1.5 T was along the first and third quadrants of the stage. Under these conditions, it implies that the semi-axis ${n}_{1}$ (Fig. 1) and the cholesteric axis will be oriented along the first–third quadrants of the microscope stage. The cholesteric pitch was measured for the Ch_D_ phase (35 °C) from microscopic texture using a microscope stage micrometer calibration slide with a resolution of 0.01 mm as $150\pm 10 \mu \mathrm{m}$.

Initially, the samples were kept at 35 °C in the presence of a magnetic field to obtain well-oriented N_D_ and Ch_D_ phases. Then, the samples were cooled at 0.05 °C/min until reaching the N_C_ and N_C_^*^ phases, respectively. During this process, microscopic textures were recorded with a Full HD Camera coupled to the microscope at a rate of 0.02 °C/photo.

The refractive indices along the N_D_–N_B_–N_C_ phase sequence were determined through the Abbe Refractometer 3 T Atago, with an accuracy of $\pm 0.0002$. Details of the experimental procedure for determining the ordinary and extraordinary refractive indices of the uniaxial nematic phases (N_D_ and N_C_), and the refractive indices ${n}_{1}$ and ${n}_{3}$ of the biaxial nematic phase can be found in the Refs. [22, 52].

By the Abbe refractometer, only two refractive indices can be obtained for the N_B_ phase. The third refractive index, ${n}_{2}$, can be calculated from the birefringence data $\Delta {n}_{21}$, which can be obtained from the Berek compensator [53] or laser conoscopy [54] techniques. In this work, the Berek compensator technique was employed to obtain $\Delta {n}_{21}$ and, consequently, ${n}_{2}=\Delta {n}_{21}+{n}_{1}$ was determined. The procedure followed here is well described in Refs. [22, 53]. To reinforce our results, the laser conoscopy was also employed in the present work to determine the birefringences $\Delta {n}_{21},\Delta {n}_{32},$ and $\Delta {n}_{31}$. The experimental procedure of this technique can be viewed in Refs. [55, 56].

The procedures reported in Refs. [57–59], by which the Michel-Lévy color chart was simulated by computer, were considered in this work to simulate the cholesteric textures. In summary, the birefringence given by Eq. (5) was used in Eq. (2) to obtain the spectrum $I$ of the light transmitted over the range of visible wavelengths of light. The spectrum $I$ was obtained considering two full rotations of the nematic plane around the cholesteric axis, i.e., two pitch lengths. In the sequence, the tristimulus values of the XYZ color space of CIE (International Commission on Illumination) were used to calculate the perceived colors of the light transmitted. Finally, the tristimulus values XYZ were converted into RGB values to display the colors (microscopic textures) on a computer monitor. The standard spectral illuminant A, defined by CIE, which is associated with the light spectrum of a halogen lamp, was also taken into account in the procedure. In addition, gamma correction was considered equal to one.

## Results

### Refractive index and birefringence measurements

**Fig. 2:**
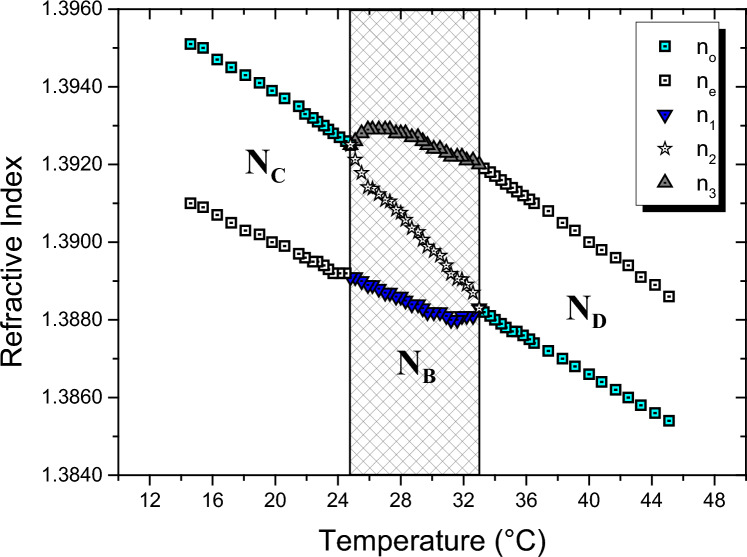
Refractive index measurements along the N_C_–N_B_–N_D_ phase sequence. The highlighted area in the figure corresponds to the temperature range of the biaxial nematic phase. The estimated uncertainty for the reading of the refractive index is 0.0002

Figure 2 shows the refractive indices measured along the N_C_–N_B_–N_D_ phase sequence using the Abbe refractometer and Berek compensator. It can be observed in the N_C_ and N_D_ phases that the refractive indices decrease monotonically with increasing temperature. At the N_C_–N_B_ phase transition, the component of the ordinary refractive index $({n}_{o})$ splits into ${n}_{3}$ and ${n}_{2}$ ones. However, the extraordinary refractive index $({n}_{e})$ remains unchanged in the N_B_ phase, and it is given by ${n}_{1}$. Locally, ${n}_{3} ({n}_{1})$ shows a maximum (minimum) in the vicinity of the N_C_–N_B_ (N_B_–N_D_) phase transition, while ${n}_{2}$ only decreases faster until it reaches the component ${n}_{1}$ approaching the N_B_–N_D_ phase transition. It is also observed in Fig. 2 that when the N_B_ phase transits to the N_D_ one, the refractive indices ${n}_{2}$ and ${n}_{1}$ join into a unique index, denoted now by ${n}_{o}$. The pattern of these refractive indices is similar to that obtained for another lyotropic mixture exhibiting the same phase sequence [22]. On the other hand, in Refs. [52, 60], a local minimum in ${n}_{1}$ was not observed by the authors near the N_B_–N_D_ phase transition.

**Fig. 3:**
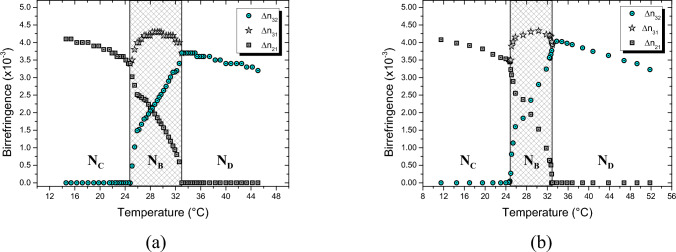
Birefringence measurements via Abbe refractometer (**a**) and laser conoscopy (**b**) along the N_C_–N_B_–N_D_ phase sequence. The estimated uncertainties for the birefringences are **a** $0.3\times {10}^{-3}$ and **b** $0.01\times {10}^{-3}$

The birefringences ($\Delta {n}_{31}$, $\Delta {n}_{32}$ and $\Delta {n}_{21}$) were determined from the refractive index data given in Fig. 2, and they are shown in Fig. 3a. In addition, Fig. 3b shows the birefringences obtained directly from the laser conoscopy technique. It can be observed that they are in good agreement with each other, and besides, they are also in agreement with other results in the literature [50, 54]. In this work, it is important to remember that only the patterns of the birefringences $\Delta {n}_{21}$ and$\Delta {n}_{31}$, as shown in Fig. 3, are essential for interpreting the colors of the cholesteric stripes, which will be discussed later.

### Simulated lyotropic cholesteric textures

**Fig. 4:**
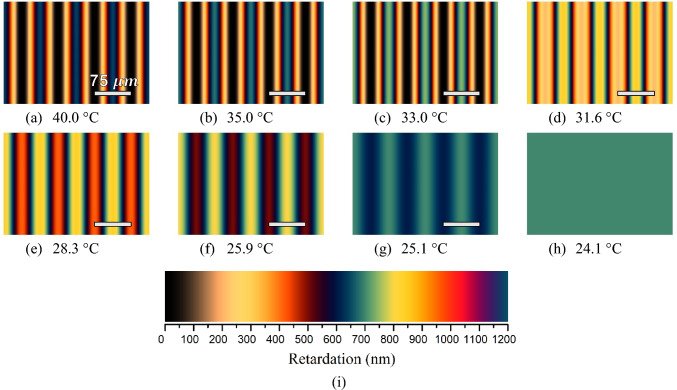
Microscopic textures simulated along the Ch_D_–Ch_B_–N_C_^*^ phase sequence, considering a pitch length $(P)$ of 150 $\mu \mathrm{m}$. The total length of the images corresponds to $2P$, and a scale bar with half pitch is shown in all cholesteric textures. **a** and **b** Ch_D_ phase; **c** Ch_B_ in the vicinity of the Ch_D_–Ch_B_ phase transition; **d**–**f** Ch_B_ phase; **g** Ch_B_ in the vicinity of the Ch_B_–N_C_^*^ phase transition; **h** N_C_^*^ phase (unwound Ch_C_ phase by the effect of the magnetic field); **i** Michel-Lévy color chart

Considering the refractive indices data of Fig. 2 and a sample with a thickness $(d)$ of 200 $\mu$m, the interference colors of the oriented textures observed under a polarized microscope along the Ch_D_–Ch_B_–N_C_^*^ phase sequence were simulated theoretically. The results are shown in Fig. 4. It is essential to note that the simulation encompassed two full rotations of the indicatrix (nematic plane) around the cholesteric pitch axis, and a pitch length of 150 *μ*m, which is the same length obtained experimentally by microscopy, was assumed to generate the microscopic textures. In our experimental studies, it was determined that the pitch length did not significantly change or change slightly within the studied temperature range in the same cholesteric phase region, for example, in the Ch_D_ phase, as reported in the literature [38]. Furthermore, the size of the pitch only causes the rotation of the indicatrix around the cholesteric axis to be faster or slower, according to a short or long pitch length, respectively. In the latter case, the interference colors along one stripe are affected with faster or slower changes, i.e., the colors rise and fall rapidly along the Michel-Lévy color chart. It is also important to notice that the choice of 200 $\mu$m of sample thickness in the simulated and experimental studies is not arbitrary. It is well known from the literature that two parameters control the transformation of the nematic phase to the cholesteric phase by doping the nematic host mixture with a chiral dopant or unwinding the helical structure of the cholesteric phase: temperature and external magnetic or electric field [61]. In the case of the constant temperature, the external magnetic field is the control parameter for the unwinding of the helix. If the external magnetic field acts as an additional control parameter, a more precise control parameter is the confinement ratio, ${C}_{R} =d/P$. For ${C}_{R}\le ({C}_{C}\approx 1.2)$, where ${C}_{C}$ is the critical confinement ratio, the helix structure of the cholesteric phase is unwound. In other words, the value of ~ 1.2 is the minimum value for C_R_ to observe the cholesteric stripes in the polarizing optical microscopy measurements. In our case, we measured the cholesteric pitch length as 150 $\mu \mathrm{m}$ at a constant temperature (35 °C), and the value of C_R_ is 200 $\mu \mathrm{m}$/150 $\mu \mathrm{m}$ = 1.33. We could not use a 100 $\mu \mathrm{m}$ sample thickness because, in this case, C_R_ = 0.67 (< 1.2). Furthermore, we could not use a sample thickness of 300 $\mu \mathrm{m}$ (or thicker) because C_R_ $\ge$ 2.00, and, as it was reported, a fingerprint texture is still observed for C_R_ ≥ 1.6, but the fingers adhere together. Consequently, the optimum sample thickness was determined as 200 $\mu \mathrm{m}.$

In Fig. 4a and b, colored and black stripes can be observed; this pattern is characteristic of Ch_D_ textures [29–31, 45]. The black and colored stripes indicate that the optic axis of the N_D_ phase is homeotropic and parallel to the stage of the optical microscope, respectively. Between the middle of the black and the middle of the blue stripes, the optic axis of the N_D_ phase suffers a rotation of 90°; consequently, the birefringence increases from zero to a maximum value given by $\Delta {\mathrm{n}}_{31}$. The interference colors between these two extremes follow the Michel-Lévy color chart, starting with zero retardation (black color) and going to a maximum retardation (blue color), where the last color is given by $R=d\Delta {n}_{31}$*.*

The simulated texture of the Ch_B_ phase immediately following the Ch_D_–Ch_B_ phase transition is shown in Fig. 4c. It appears that the black stripe remains unchanged, but indeed, this is incorrect. It is almost black because the birefringence $\Delta {n}_{21}$ is still very small (~ 10^–4^). On the other hand, the blue stripe has become slightly lighter due to a faster increase in the birefringence $\Delta {n}_{31}$, Fig. 3.

From Fig. 4c–g, the lighter blue color increases to a yellow color in the Michel-Lévy color chart along the Ch_B_ phase. It is a result of a slight increase in $\Delta {n}_{31}$, and it remains almost constant in the N_B_ phase, see Fig. 3. When the Ch_B_ approaches the Ch_C_ phase, the color of this stripe decreases to a pale yellow and then to a blue-green color, as shown in Fig. 4g. On the other hand, the “black” color stripe increases faster than the previous one because $\Delta {n}_{21}$ highly depends on temperature; it always increases with temperature, as shown in Fig. 3. Furthermore, the interference color of this stripe increases along the Michel-Lévy color chart until a dark blue color is observed, Fig. 4g. Besides this, in the Ch_B_ phase, it is also possible to observe a “black” thin line inside the colored stripes, Fig. 4c–e. This line corresponds to a first-order retardation in the Michel-Lévy chart; therefore, the “black” color is actually an interference color between deep red and indigo colors. Note that this thin line becomes less pronounced as the birefringence varies with temperature. In addition, the black color cannot exist completely in the Ch_B_ phase; it exists locally on each biaxal nematic plane with two optic axes whose directions do not coincide with a direction normal to the microscope stage [43].

Going from Fig. 4g–h, it is observed that the stripes vanish at the Ch_B_–Ch_C_ phase transition. Here, the helicoidal structure of the Ch_C_ phase is unwound by the applied magnetic field, transforming it into the N_C_^*^ phase [22, 30, 31, 39–41], which is equivalent to the N_C_ one [41, 62]. Finally, a uniform color of the oriented texture of the N_C_^*^ phase can be observed in Fig. 4h. The relation between interference colors of Fig. 4a–h and retardation $(R)$ can be obtained from the Michel-Lévy color chart shown in Fig. 4i.

**Fig. 5:**
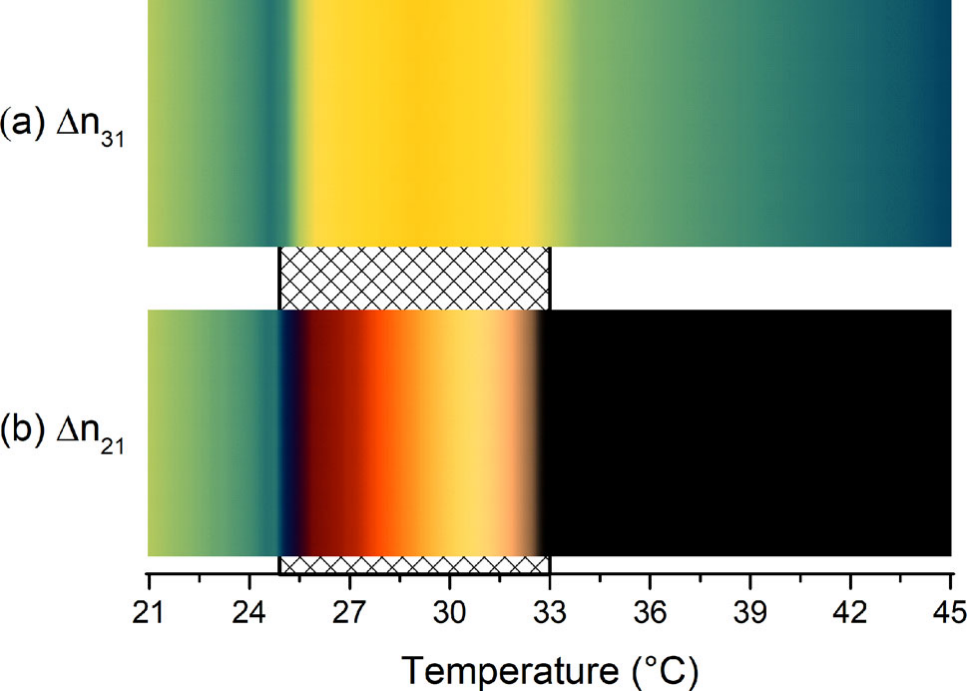
Simulated interference colors associated with two nematic planes along the cholesteric phase sequence: Ch_D_–Ch_B_–N_C_^*^. Interference colors associated with the birefringence $\Delta {n}_{31}$ and $\Delta {n}_{21}$ are shown in (**a**) and (**b**), respectively

Now, considering only the middle of the stripes defined by the birefringences $\Delta {n}_{31}$ and $\Delta {n}_{21}$, i.e., for two specific nematic plane orientations, the interference colors along the Ch_D_–Ch_B_–N_C_^*^ phase sequence were simulated by interpolating the birefringence values of Fig. 3a at intervals of 0.1 °C. The results obtained for $\Delta {n}_{31}$ and $\Delta {n}_{21}$ are shown in Fig. 5a and b, respectively. These help us to understand and to visualize how the colors (Retardation, $R$) of the stripes change along the Ch_D_–Ch_B_–N_C_^*^ phase sequence. From Fig. 5, it is essential to note that both birefringences increase steeply near the Ch_D_–Ch_B_ phase transition. On the other hand, the birefringence $\Delta {n}_{21} (\Delta {n}_{31})$ increases (decreases) faster near the Ch_B_–N_C_^*^ phase transition until they reach the same value. Besides, the Ch_B_ phase is comprehended by two imaginary vertical lines that contrast two distinct colors associated with a little discontinuity, i.e., a “jump”, in the colors of the Michel-Lévy color chart, Fig. 5a and b.

From Fig. 5b, it is also essential to note that this result is in good agreement with Ref. [49]. Here, it can be seen that the nematic plane of the cholesteric phases associated with the birefringence $\Delta {n}_{21}$ represents the same behavior of the oriented nematic phase sequence, N_D_–N_B_–N_C_, obtained for the same lyotropic mixture without the addition of the chiral dopant (brucine). This was an expected situation because, as was shown in Ref. [31], brucine does not modify the micelle dimensions; instead, it acts on the micelles to twist them around the helix axis.

### Experimental lyotropic cholesteric textures

**Fig. 6:**
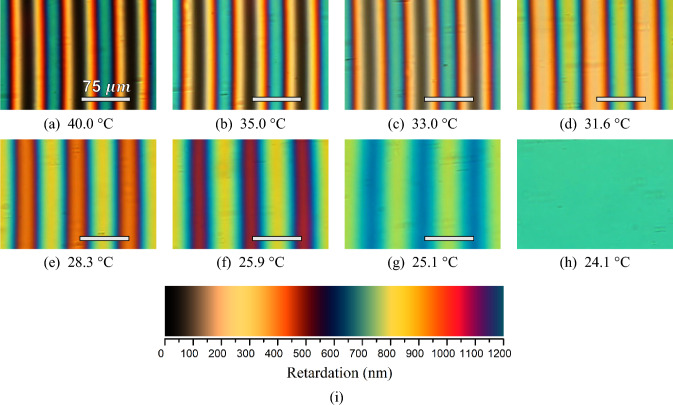
Experimental microscopic textures obtained under crossed polarized microscopy along the Ch_D_–Ch_B_–N_C_^*^ phase sequence. The experimental pitch length $(P)$ measured is about 150 $\mu \mathrm{m}$, a scale bar with half pitch is shown in all cholesteric textures. **a** and **b** Ch_D_ phase; **c** Ch_B_ near of Ch_D_–Ch_B_ phase transition; **d**–**f** Ch_B_ phase; **g** Ch_B_ near of Ch_B_–N_C_^*^ phase transition; **h** N_C_^*^ phase (Ch_C_ phase unwounded by the magnetic field); **i** Michel-Lévy color chart

The oriented microscopic textures of the Ch_D_–Ch_B_–N_C_^*^ phase sequence, obtainedfrom the polarizing microscope investigations, with the optic axis of the cholesteric phases forming an angle that bisects the angle between the polarizer and the analyzer, are shown in Fig. 6.

Figure 6a and b shows the textures obtained for the Ch_D_ phase at 40 °C and 35 °C, respectively. Note that these textures are in agreement with the Ref. [31], and they also exhibit a good concordance with the simulation, Fig. 5a and b. Figure 6c shows the texture of the Ch_B_ phase just after the Ch_D_–Ch_B_ phase transition. Here, it is possible to observe that thin stripes with yellow color start to appear in the middle of the blue stripes. It means that, after the Ch_D_–Ch_B_ phase transition, the retardation starts to increase, which is in accordance with the birefringence $\Delta {n}_{31}$, Fig. 3. On the other hand, it is observed that the black stripes cause the appearance of the region with brownish color at the Ch_D_–Ch_B_ phase transition [31, 45]. It occurs because $\Delta {n}_{21}$ becomes different than zero in the Ch_B_ phase, and it increases quickly until the phase approaches the Ch_C_ one. In addition, it can be seen in Fig. 6c–g that the brownish color increases until a dark blue color along the Ch_B_ phase, following the Michel-Lévy color chart, while the yellow color remains almost unchanged. The changes in the colors of the Ch_B_ stripes arise from the changes in the $\Delta {n}_{31}$ and $\Delta {n}_{21}$ birefringences of Fig. 3. Lastly, the texture of the N_C_^*^ phase (Ch_C_ unwound by the magnetic field) with a uniform blue-green color is shown in Fig. 6h. Note that the color of Fig. 6h seems to match the colors of Fig. 5h, with the former lighter than the latter. Figure 4i shows the Michel-Lévy color chart for a quick comparison between the interference colors of Fig. 5a–h and the values of the retardation $(R)$.

At this point, it is important to emphasize that when the experimental textures (Fig. 6) are compared with the simulated ones (Fig. 5), some differences between them can be observed, which can be associated with the quality of the camera used to capture the microscopic textures, and its configuration (e.g., saturation, color corrections, brightness, and exposure) may contribute to these discrepancy differences between the experimental and theoretical results. Furthermore, we should expect that other factors may also contribute to it, for instance, the thickness of the sample and the real spectral light bulb of the microscope lamp. In addition, differences in the interference colors can also be observed for two experimental results, Fig. 6 of the present study and Fig. 1 of Ref. [31]. Nevertheless, these results give us an understanding of the interference colors presented by oriented Ch_D_–Ch_B_–N_C_* phase sequence. To the best of our knowledge, this interpretation of the interference colors of the microscopic textures in cholesteric phases, considering both refractive indices and birefringences, has not been made in the literature yet. In this context, further work may be needed in the future to address these differences.

## Conclusions

The lyotropic nematic phase sequence, N_D_–N_B_–N_C_, was characterized by refractometry and laser conoscopy techniques, which showed agreement with other results previously published in the literature. From these results, the microscopic textures of the oriented cholesteric phase sequence, Ch_D_–Ch_B_–N_C_^*^ (Ch_C_ unwound by the magnetic field), were simulated theoretically, and the interference colors of each stripe were interpreted and discussed. Besides, when the simulated cholesteric textures were compared with the experimental cholesteric textures, a little difference between the colors of the stripes was observed. It was associated with the quality of the digital camera, its configuration, and other factors.

## Data Availability

The data supporting the findings of this study are available within the article and they can be made available on reasonable request.
